# Dimension-based retro-cue benefit in working memory does not require unfocused dimension removal

**DOI:** 10.3389/fpsyg.2024.1433405

**Published:** 2024-11-01

**Authors:** Ruyi Liu, Lijing Guo, Xiaoshu Lin, Dan Nie, Piia Astikainen, Chaoxiong Ye

**Affiliations:** ^1^School of Education, Anyang Normal University, Anyang, China; ^2^Institute of Brain and Psychological Sciences, Sichuan Normal University, Chengdu, China; ^3^Department of Psychology, University of Jyvaskyla, Jyväskylä, Finland; ^4^Faculty of Medicine, University of Helsinki, Helsinki, Finland

**Keywords:** visual working memory, dimension-based attention, retro-cue benefit, double retro-cues, visual short-term memory

## Abstract

**Introduction:**

Within the maintenance phase of visual working memory (VWM), previous researchers presented retro-cues orienting to a probed dimension across all multidimension stimuli and found a robust dimension-based retro-cue benefit (RCB): VWM performance for cued dimension was better than no/neutral-cue baseline. This improvement is often attributed to the prioritization of information related to the focused dimension and the removal of information related to the unfocused dimension from VWM. However, it remains unclear whether the removal of the uncued dimension is necessary to observe this dimension-based RCB.

**Methods:**

In the current study, we first manipulated the number of retro-cues to investigate this question. We used colored, oriented bars as stimuli and two sequential retro-cues oriented to different dimensions in the double-cue condition. The last presented cue in each trial was always valid. Therefore, the unfocused dimension in the first cue display was probed in double-cue trials. Experiment 1 adopted change detection tasks and three cue type conditions (no-cue, single-cue, double-cue). Experiment 2 divided the single-cue condition into early- and late- cue conditions, using recall tasks to elevated probe precision. Experiment 3 further added double-neutral and double-same cue types and eliminated the different influences of post-memory masks on each dimension respectively.

**Results:**

Results across these experiments showed a robust pattern of no worse performances for the double-cue condition than for the single-cue condition.

**Discussion:**

Because the dimension-based single cue benefit was observed especially in early-cue trials, we supposed that the dimension-based RCB does not require removing the unfocused dimension from VWM.

## 1 Introduction

Visual working memory (VWM) serves as a temporary online platform for encoding, maintaining, and retrieving visual information (Baddeley, 2012; Geigerman et al., 2016; Luck and Vogel, 2013). VWM is closely linked to various other cognitive functions, including attention (Kong and Fougnie, 2019), long-term memory (Fukuda and Vogel, 2019; Fukuda and Woodman, 2017; Hartshorne and Makovski, 2019), and decision-making (Schapiro et al., 2022). Consequently, the limited capacity and flexible task-driven processes of VWM have garnered significant research interest over the past two decades (Gao et al., 2011; Liu et al., 2022; Luck and Vogel, 1997; Luria and Vogel, 2011; Vogel and Awh, 2008; Ye et al., 2014; Ye et al., 2020; Ye et al., 2019; Ye et al., 2017; Long et al., 2020).

Attention plays a crucial role in guiding the allocation of resources among the representations within VWM. When faced with filtering tasks involving targets and distractors, individuals utilize attention to enhance the salience of task-relevant objects while suppressing distractors from entering the VWM platform (Duan et al., 2023; Hakim et al., 2021; Hakim et al., 2020; Qi et al., 2014; Ye et al., 2024; Ye et al., 2024; Ye et al., 2023; Liu et al., 2024). This process often relies on object-based attention, which is influenced by the spatial location of objects. When features from different dimensions (such as color and shape) are integrated into the same object, they are attended to holistically. The object-based attention is typically directed by spatial cues (e.g., an arrow pointing to the targets) or feature cues (e.g., in a face memory task, faces with red borders as targets and those with yellow borders as distractors) in laboratory tasks (Luck and Vogel, 1997; Ye et al., 2018). In addition to the behavioral task, Vogel et al. (2005) have also used electroencephalogram (EEG) to demonstrate how object-based attention effectively prevents distractors from accessing VWM.

In addition to the object’s location, or contextual information, dimension cues also direct attention. In search tasks involving stimuli with varying colors and shapes, the dimension-based attention selectively focuses on features from a specific dimension across all objects (e.g., the color information of all objects), while features from other dimensions (e.g., shape information) exert minimal disruption on the search efficiency (Bacon and Egeth, 1994; Krummenacher and Muller, 2012; Theeuwes, 1992). When tracking moving objects that change colors, individuals can flexibly use object-based or dimension-based attention to ignore irrelevant information and track the targets (Hopf et al., 2005; Schoenfeld et al., 2003). However, the storage of information in VWM is tightly bound to context, which poses a challenge for the selection of dimension-based attention (Gao et al., 2016; Shen et al., 2013; Woodman and Vogel, 2008). Shen et al. (2013) explored this by manipulating the task relevance of a changed dimension within a probe array during a memory task. They found that changes in shapes, which participants were instructed to ignore, slowed the detection of target colors. This finding indicated that the object-based process in VWM hinders dimension-based attention from fully excluding irrelevant dimensions from the VWM platform. Therefore, the selective advantages of object-based and dimension-based attention are contingent upon the intrinsic storage characteristics of VWM.

To the researchers’ surprise, dimension-based attention exerts a remarkably strong and positive influence on the relevant dimensions of VWM representations during the maintenance phase (Heuer and Schubö, 2017; Liu et al., 2023; Ye et al., 2021; Niklaus et al., 2017; Park et al., 2017; Ye et al., 2016; Hajonides et al., 2020). Previous studies used recall tasks in which participants were required to memorize objects with multiple dimensions and subsequently select the probed color or orientation from a wide array of options. To direct participants’ dimension-based attention, researchers provided dimensional cues indicating the dimension to be probed during the memory–probe interval. As a result, the participants utilized these retro-cues to reallocate their attention and process the relevant representations online. Consistent findings across these studies revealed that VWM performance for the dimension highlighted by valid cues was significantly better than in neutral or no-cue baseline conditions, a phenomenon referred to as the dimension-based retro-cue benefit (RCB). Conversely, compared to the baseline, participants exhibited worse memory performance when the invalid cue condition probed the uncued, unfocused dimension, a phenomenon known as the dimension-based retro-cue cost. Accordingly, Ye et al. (2016) proposed that the observed dimension-based RCB or costs arise from a dual mechanism. First, the retro-cue allows for the prioritization and protection of the focused dimension, which reduces the decay of its representation during subsequent maintenance and retrieval processes. Second, the retro-cue frees up valuable VWM resources by withdrawing cognitive resources from the unfocused dimension, thereby minimizing competition between the focused and unfocused dimensions.

However, to our knowledge, few studies on the dimension-based RCB have thoroughly examined the necessity of removing information related to the unfocused dimension. Previous studies used an invalid-cue condition and found the dimension-based retro-cue cost (Niklaus et al., 2017; Park et al., 2017). Two explanations might account for this phenomenon. First, participants might actively remove the information related to the unfocused dimension from their VWM, leading to complete forgetting of the unfocused dimension. Alternatively, due to the limited attentional resources being reallocated to the cued dimension, the unfocused dimension in VWM could become more susceptible to disruption by internal competition among representations and interference from irrelevant external inputs. As a result, retro-cue studies using a single cue cannot definitively determine whether the unfocused dimension in VWM is actively removed or simply disrupted; nor can they ascertain whether removing the unfocused dimension is essential for achieving the dimension-based RCB. Additionally, previous studies have utilized a mixture model analysis to assess memory precision and guess rates (Park et al., 2017; Zhang and Luck, 2008). Notably, even under invalid-cue conditions with a high memory load, guess rates remain relatively low, suggesting that participants may not completely eliminate the unfocused dimension from their VWM. Moreover, it remains unclear whether participants’ decisions to abandon a dimension in VWM can be reliably inferred from guess rates alone. Therefore, considering the dimension-based retro-cue cost observed in VWM tasks with a single retro-cue and the results of mixture model analyses, we cannot definitively conclude whether the dimension-based RCB requires the removal of the unfocused dimension from VWM.

In this present study, we used VWM tasks with double retro-cues to guide attention across dimensions in VWM and to investigate the necessity of removing the unfocused dimension. A key experimental manipulation was the number of retro-cues. In the single retro-cue condition, only one retro-cue was presented, and it was always valid. In the double retro-cue condition, participants were presented with two successive retro-cues directed at different dimensions, with the last cue always being valid. This design implied that the first cue in the double-cue trials was invalid. However, participants could not ignore the first cue following the memory array, as all cue types were intermixed, preventing them from judging the validity of each cue. Therefore, the most economical strategy would be to maintain the unfocused dimension in VWM for a potential probe after the first cue. If participants performed better in the single retro-cue condition compared to the no-cue baseline, it would indicate that they used the first cue to focus attention on the corresponding dimension. Under this scenario, if performance in the double retro-cue condition also exceeded the baseline, it would suggest that the unfocused dimension was not removed from VWM after the first cue. This is because even if the second cue guided attention back to the unfocused dimension, the removed information could not be recovered. Therefore, these results suggest that the removal of the unfocused dimension would be necessary to achieve the dimension-based RCB. In addition, if participants did perform badly in the double-cue condition compared to the baseline, this would suggest that they were able to shift attention to the dimension indicated by the second cue and prioritize the cued dimension information. In this case, retaining the unfocused dimension in VWM would still allow for the dimension-based RCB to emerge. Moreover, in our task, participants were motivated to retain the unfocused dimension information in VWM. If the dimension-based RCB disappeared as a result of the retention of unfocused dimension information, it would again indicate that achieving the dimension-based RCB requires the removal of the unfocused dimension information from VWM.

## 2 Experiment 1: investigating the existence of unfocused dimensions in VWM

In Experiment 1, we explored whether forgetting the unfocused dimension in VWM is a prerequisite for obtaining the dimension-based RCB. Unlike previous studies on dimension-based RCB (Niklaus et al., 2017; Park et al., 2017; Ye et al., 2016), we required participants to perform a change detection task involving double dimension-based retro-cues. Participants were instructed to memorize two colored, oriented bars and subsequently decide whether the color of a square or the orientation of a white bar matched the memorized items. In 50% of the trials, either the color or orientation in the probe array was altered. Participants responded with either “same” or “different.” An accuracy rate at chance level in the double retro-cue condition would suggest the complete elimination of the unfocused dimension following the first cue.

We manipulated three cue conditions—no-cue, single-cue, and double-cue—and used Chinese characters as cues, indicating “color” (“色,” in Chinese) or “orientation” (“向,” in Chinese). In the double-cue condition, different dimensions were sequentially cued (e.g., the first cue indicated “color,” followed by a second cue indicating “orientation”), making the probed dimension unfocused after the first cue. By comparing memory performance in the no-cue baseline condition with that in the double-cue condition, we sought to determine whether the unfocused dimension had been effectively removed from VWM during the double-cue trials.

### 2.1 Materials and methods

#### 2.1.1 Participants

Previous studies investigating the dimension-based RCB typically had 16–28 participants (Liu et al., 2023; Niklaus et al., 2017; Park et al., 2017; Ye et al., 2016). However, these previous studies primarily utilized recall tasks, whereas our Experiment 1 used a change detection task. Additionally, according to our knowledge, we were the first to use two retro-cues directed at different dimensions. Given the methodological differences between their studies and ours, we opted to recruit 26 participants to ensure an adequate sample size. For collecting 26 valid data from participants, we initially recruited 31 college students who self-reported normal health, normal color vision, and normal or corrected-to-normal visual acuity. Participants were excluded if their accuracy rate in the main task was more than two standard deviations below the mean, resulting in the exclusion of five participants. The final sample consisted of 26 participants (23 female participants, three male participants, all right-handed, mean age = 19.69 years, SD = 1.29). To ensure sufficient statistical power, we conducted a post-hoc power analysis (Faul et al., 2007) using a one-way (cue types: no-cue vs. single-cue vs. double-cue) repeated measures ANOVA design. This analysis indicated that our sample size was sufficient to detect a large effect size (η*_p_* 2 = 0.26) with 81% statistical power at a significance level of 0.05. All participants provided informed consent before the experiment and were compensated for their participation. The experimental procedures adhered to the Declaration of Helsinki (2008) and were approved by the ethical committee of Sichuan Normal University (approval number: SCNU-211228).

#### 2.1.2 Stimuli and procedure

The stimuli were selected from a pool of 360 continuous colors and 180 orientations (spanning the full 360° color space and 180° orientation range, respectively). In the memory array, each stimulus’s color and orientation differed by at least 60 color steps and 30° in orientation from any other stimulus. The color pool was generated following the method used in Ye et al. (2016), where colors were systematically varied in saturation (RGB) at each step (see more details in Supplementary material). The memory array consisted of two colored, oriented bars (length: 1.1°, width: 0.4°). In the probe array, either a colored square (1.2° × 1.2°) or a white bar (1.1° × 0.4°) was presented, corresponding to the color or orientation conditions. The experiment was programmed using E-prime 1.0 and was displayed against a gray background (RGB = 128, 128, 128) on a 21-inch LCD monitor (1,280 × 768, 75 Hz), viewed from a distance of 60 cm.

**Figure 1:**
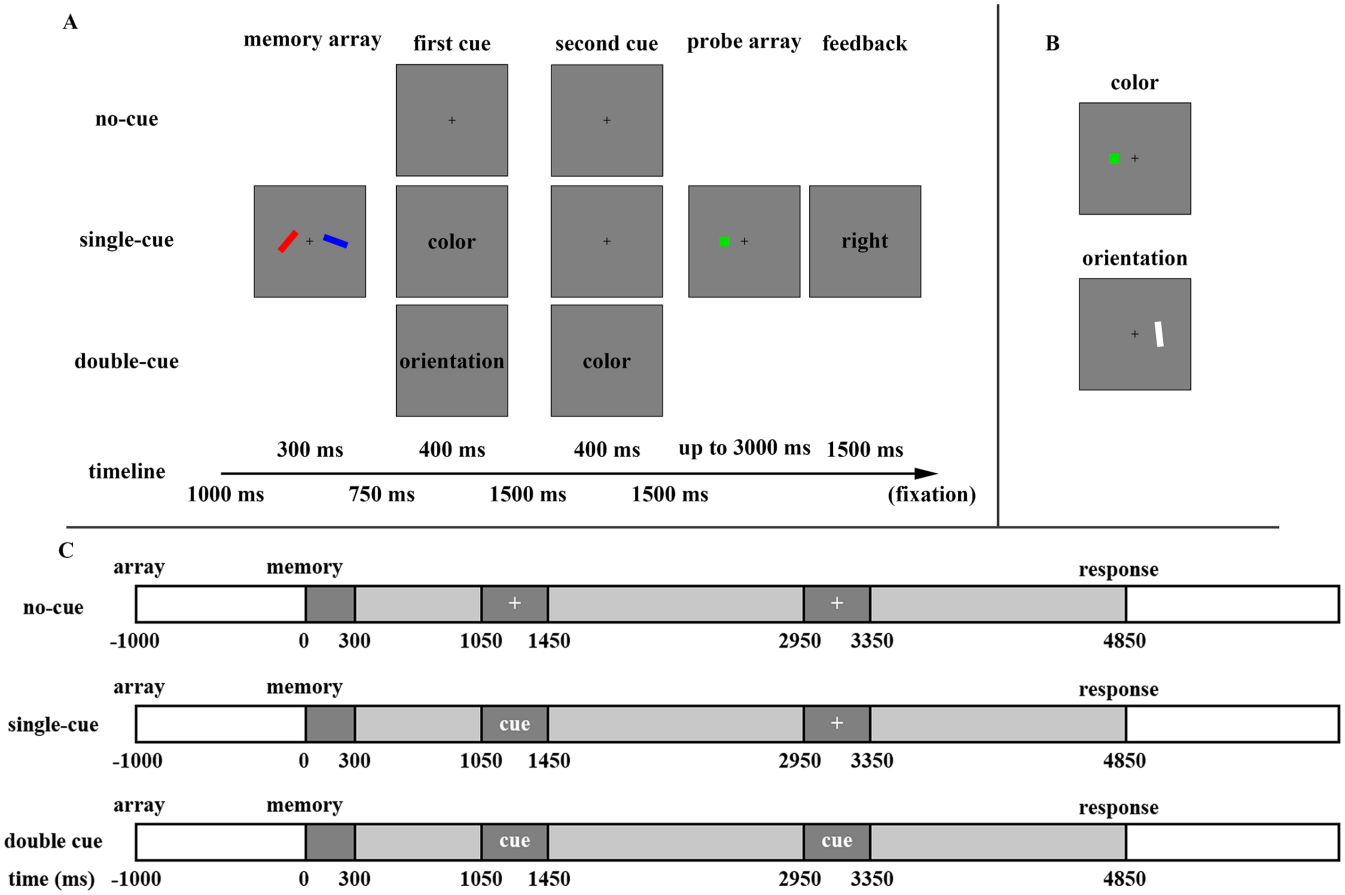
**(A)** The procedure for changed trials probing a color in the three cue-type conditions. **(B)** Examples of color and orientation probe arrays. **(C)** The time procedure for three cue-type conditions.

As shown in Figure 1, the main procedure of Experiment 1 began with a fixation cross (0.2°) centered on the screen, presented for 1,000 ms. A memory array, consisting of two colored, oriented bars positioned approximately 0.9° to the left and right of the fixation cross, was shown for 300 ms. The color and orientation of the bars varied independently, ensuring that no two identical colors or orientations appeared within the same trial. The memory array was followed by a blank, divided into three intervals (750, 1,500, and 1,500 ms, respectively) by two 400-ms cue arrays. In no-cue trials, only the fixation cross was shown in both cue arrays. In single-cue trials, a valid dimension-based cue appeared in the center of the first cue array, while the second cue array displayed only the fixation cross. In double-cue trials, different cues were shown in both cue arrays, making the first cue invalid. After the third interval, a probe array was presented for 3,000 ms (or until the participant responded). The item in the probe array occupied the same location as the corresponding item in the memory array. In 50% of the trials, the dimension of the item in the probe array matched that in the memory array, while in the remaining trials, the probed feature of color or orientation differed by at least 60 or 30 degrees, respectively, from the memory feature.

Participants were instructed to memorize the stimuli and judge whether the probed dimension in the probe array was identical to that in the memory array. They were asked to press the “F” key if no change was detected and the “J” key if a change had occurred. Emphasis was placed on using all cues, with the understanding that no more than two dimension-based cues might appear during the retention interval to aid in the memory task. Accuracy was emphasized over response speed. Feedback was provided at the end of each trial for 1,500 ms: the word “right” (“正确,” in Chinese) appeared on the screen if the participant responded correctly, while “wrong” (“错误,” in Chinese) was displayed if the response was incorrect.

Participants completed 64 trials for each cue type (no-cue, single-cue, and double-cue), resulting in 192 trials in total, following 13 practice trials. The orientation and color were probed equally across trials. The trials of three cue types were randomly intermixed. Participants were allowed to rest every 11 min during the experiment, which lasted approximately 50 min in total.

#### 2.1.3 Data analysis

Accuracy (ACC) was used as an index of VWM performance. The accuracy of color and orientation probe trials were analyzed separately using repeated-measures analysis of variance (ANOVA), with cue type (no-cue, single-cue, and double-cue) as the within-subjects factor. The partial eta squared (η*_p_* 2) value was reported as an estimate of effect size for the ANOVAs. Planned pairwise comparisons among the three cue-type conditions were conducted using paired-sample *t*-tests. Cohen’s *d* was provided by JASP software (version 0.16, JASP Team, 2021) to estimate effect sizes for the t-tests, along with Bayes factors. Specifically, we reported BF_10_ for significant results, with values greater than 3 interpreted as substantial evidence in favor of the alternative hypothesis. For non-significant results, we reported BF_01_, with values greater than 3 interpreted as substantial evidence in favor of the null hypothesis (Rouder et al., 2009; Schmalz et al., 2021).

### 2.2 Results

The results of the one-way repeated measures ANOVA for color report trials revealed a significant main effect of cue type, *F*(2, 50) = 3.261, *p* = 0.047, η*_p_* 2 = 0.115. However, the results of ANOVA for orientation report trials revealed no significant main effect of cue type, *F*(2, 50) = 0.684, *p* = 0.481, η*_p_* 2 = 0.027.

#### 2.2.1 The dimension-based single cue benefit

**Figure 2:**
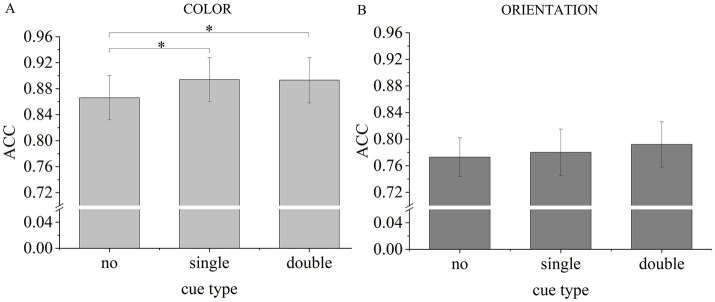
Accuracy results of Experiment 1. Light gray bars represent mean ACC for color report trials **(A)**. Dark gray bars represent mean ACC for orientation report trials **(B)**. Error bars indicate standard error (SE). *p < 0.050.

For color report trials (Figure 2A), planned comparisons revealed significantly higher accuracy rates in single-cue trials than in no-cue trials, *t*(25) = 2.466, *p* = 0.010, Cohen’s *d* = 0.484, BF_10_ = 5.046. This result indicated a robust dimension-based RCB for colors. However, for orientation report trials (Figure 2B), planned comparisons revealed no significant differences between accuracy rates in single-cue trials and in no-cue trials, *t*(25) = 0.401, *p* = 0.346, Cohen’s *d* = 0.079, BF_01_ = 3.459. This result suggested no dimension-based RCB for orientations.

#### 2.2.2 The dimension-based double cue benefit

The memory performance for colors in the double-cue condition was better than the baseline, *t*(25) = 1.929, *p* = 0.033, Cohen’s *d* = 0.378, BF_10_ = 1.972. This result evidenced that the color information in VWM had not been removed after the first cue and was prioritized by the second retro-cue. However, no significant difference was found between these conditions for orientations, *t*(25) = 1.093, *p* = 0.142, Cohen’s *d* = 0.214, BF_01_ = 1.664. This result suggested that the orientation information in VWM was maintained after the first cue, and the improvement of the second retro-cue on it was not observable in change detection tasks.

#### 2.2.3 The comparison of double-cue and single-cue conditions

Regardless of the probed dimension, participants’ accuracy rates did not differ in the double-cue trials and the single-cue trials [color: *t*(25) = 0.157, *p* = 0.562, Cohen’s *d* = 0.031, BF_01_ = 5.414; orientation: *t*(25) = 0.959, *p* = 0.173, Cohen’s *d* = 0.188, BF_01_ = 1.950]. These results suggested that the prioritization of focused attention on information in VWM might be independent of attention shift times.

### 2.3 Discussion

In Experiment 1, we used a change detection task with double-dimension memory stimuli and manipulated the number of retro-cues. VWM performance in double-cue trials was not worse than in trials without any cues, particularly when the probed dimension was color. Because the absence of a significant difference between these conditions for orientation reports could be considered inconclusive based on Bayes factor results, we could not infer whether orientation performance in double-cue trials was better or worse than the baseline condition. Thus, our findings suggest that participants did not remove the unfocused dimension (at least when the unfocused dimension was color) from VWM following the first cue display.

We only observed a dimension-based RCB for color, but not for orientation. It is possible that when the first cue indicated “color,” attention was preferentially allocated to the color information, protecting this memory from decay or interference from the test array, which led to better performance in single-cue trials compared to no-cue trials. We speculated that the remaining cognitive resources were sufficient to maintain orientation information at a level comparable to the no-cue baseline, as indicated by the orientation performance in double-cue trials not being worse than the baseline. Regarding the use of orientation cues, we propose two possible explanations. First, the necessity of removing unfocused dimension information may differ between color and orientation. When participants saw the first orientation cue, the anticipation of a subsequent color cue may have discouraged them from removing color information from VWM, thereby hindering the enhancement of focused orientation memory in VWM. Consequently, in the change detection task, orientation retro-cues might require more VWM resources than color retro-cues, consistent with our finding that the dimension-based double cue benefit was significantly observed for color but not for orientation (Liu et al., 2023). Previous studies on VWM consolidation have also demonstrated differing bandwidth demands between color and orientation (Miller et al., 2014). Second, due to the differing difficulty levels in matching color-probed and orientation-probed arrays in the change detection task, it is possible that participants primarily utilized the color retro-cue, focusing their attention exclusively on color cues and maintaining this allocation even when presented with orientation cues.

Interestingly, there was no significant difference between performance for focused colors in single-cue trials and refocused colors in double-cue trials, suggesting that the number of attention shifts did not diminish the benefit gained from focusing attention on color information. However, this finding should be interpreted with caution, as participants may not have used orientation retro-cues to guide their attention. In the General Discussion, we will further discuss whether dimension-based attention shifts result in any potential costs to VWM performance.

In Experiment 1, we observed no new evidence for a dimension-based RCB for orientations in the change detection task. This result may stem from the paradigm used in Experiment 1, specifically the change detection task with double retro-cues. Most previous studies that identified the dimension-based RCB used recall tasks as the VWM tasks (Liu et al., 2023; Niklaus et al., 2017; Park et al., 2017; Ye et al., 2016). The change detection task may be less sensitive to the dimension-based retro-cue effect, making it more challenging to detect the removal of unfocused color information from VWM. The limited probe precision inherent to change detection tasks could contribute to this lack of sensitivity. For example, in Experiment 1, the attribute change in the probe array was at least 60 degrees for colors and 30 degrees for orientations compared to the memory feature. When the probe stimulus involved orientation, the difficulty of judging whether the target stimulus matched the probe might have been higher than when the probe stimulus involved color. Participants may have strategically opted to use retro-cues only for color, leaving the color information in VWM consistently focused throughout the task. Subtle differences among cue types may have been obscured by the binary response format of the change detection task. To increase probe precision, VWM studies have typically narrowed the degree of change between stimuli, either by selecting more similar memory and probe stimuli in change detection tasks (Lin and Luck, 2009) or by using recall tasks that require participants to choose a precise level from 180/360 options (Zhang and Luck, 2008). Nevertheless, the change detection task’s binary response format inherently limits the capture of fine details in participants’ memorized representations. Moreover, our previous research found that the dimension-based RCB was weaker in the change detection task but more pronounced in the recall task (Liu et al., 2023).

Additionally, in Experiment 1, we presented two bars at fixed locations in the memory array (Niklaus et al., 2017), facilitating orientation grouping. While previous research has shown that grouping aids VWM (Allon et al., 2018; Kaiser et al., 2014; Chen et al., 2021), these studies typically included both targets and non-targets in the probe array. Participants may need to see the non-target grouped with the target to utilize the memorized integration effectively in their decision-making (Prieto et al., 2022). However, Experiment 1 only presented the target orientation in the probe array. Without a reference orientation on the screen, participants had to use relative information within the integrated representation for orientations while resisting interference from the non-target orientation. Thus, orientation grouping might have inadvertently hindered decision-making in orientation change detection tasks.

Taken together, the results of Experiment 1 suggest that the unfocused dimension may not be removed from VWM when the dimension-based RCB is present. However, this conclusion is tentative due to potential participant cue preferences and the low probe precision inherent in change detection tasks.

## 3 Experiment 2: examining the existence of unfocused dimensions with a high-probe precision task

In Experiment 1, we used a change detection task and manipulated the number of retro-cues to examine the necessity of removing unfocused dimensions in the dimension-based RCB within VWM. Our results suggest that the unfocused dimension may not be removed when focused attention prioritizes the memory of another dimension in VWM. However, we did not observe a dimension-based RCB for orientations, indicating potential differences in participants’ motivation for utilizing cues for different dimensions. The low probe precision inherent in change detection tasks might have obscured differences between cue types for probed orientations in Experiment 1. Participants’ strategies, such as exclusively relying on color cues, may also have diminished the effectiveness of orientation cues.

To avoid the potential limitation from the change detection task, in Experiment 2, we used a recall task as the VWM task as described in previous studies (Liu et al., 2023; Niklaus et al., 2017; Park et al., 2017; Ye et al., 2016), which provided options of 360 colors or 180 orientations in probe arrays. This task allowed us to analyze participants’ performances with greater precision and detect subtle differences between conditions. Furthermore, using a recall task helped reduce potential interference from other factors introduced by the probe arrays in the change detection task. Additionally, we asked participants to memorize three colored, oriented bars at three locations randomly chosen from four options in each trial, increasing the difficulty of orientation grouping (Ye et al., 2016). Furthermore, the demand for detailed memory in recall tasks, combined with the increased memory load, likely heightened participants’ motivation to use the retro-cue. We also manipulated more complex cue types (neutral-cue, early-cue, late-cue, and double-cue) compared to Experiment 1. Notably, in neutral-cue and late-cue trials, we presented neutral cues (the word “all,” “全” in Chinese) in the first cue display. Neutral cues indicated that any dimension could be probed and did not confer any specific benefit for VWM of a particular dimension. We used neutral cues in the baseline condition (neutral cue condition) rather than fixation crosses as Experiment 1 to eliminate the potential noise caused by the sudden appearance of words, regardless of their content. To address the possibility that participants might only apply a cue once they were certain it was the final one, we established two single-cue conditions: early cue and late cue. In late-cue trials, a neutral cue appeared in the first cue array, followed by a dimension-based cue in the second-cue array. In early-cue trials, a dimension-based cue was presented in the first cue array, with the probe array appearing at the same time as the second cue in late-cue and double-cue trials. Since participants require time to allocate attention according to retro-cues (Ye et al., 2016), if they only used the late-cue, the probe array in early-cue trials would not allow sufficient time for attention deployment, resulting in the absence of a dimension-based RCB.

To prevent participant fatigue, we redesigned the duration of Experiment 2 by reducing the display times for the cue and post-cue intervals, producing a cue-to-probe SOA of 1,100 ms, which did not affect participants’ ability to identify cues and allocate attention (Liu et al., 2023; Park et al., 2017). We also included masks following the memory array to minimize the afterimage of the memory array, as done in previous VWM studies (Becker et al., 2013; Hao et al., 2018; Xie and Zhang, 2017; Ye et al., 2024). This design helped exclude the influence of iconic memory on task performance, especially in the early-cue condition, where focused attention might select dimensional information from iconic memory.

### 3.1 Materials and methods

#### 3.1.1 Participants

Given the absence of dimension-based RCBs and the findings for orientations observed in Experiment 1, we aimed to address these issues by slightly increasing our sample size to 31 in Experiment 2. A new cohort of 35 college students was recruited under the same criteria as in Experiment 1. However, one participant was excluded due to the program crash, and three additional participants were excluded due to excessively high reproduction errors for each probed dimension in the neutral-cue condition (exceeding 2 standard deviations above the mean) during the recall task. This resulted in a final sample of 31 participants (one left-handed, 27 female participants, four male participants, mean age = 20.45 years, SD = 1.52) for further analysis. The sample size was deemed sufficient to detect a large effect size (η*_p_* 2 = 0.26) with 93% power at a significance level of 0.05, as determined by a power analysis (Faul et al., 2007) using a one-way (four levels) repeated measures ANOVA design. All participants provided informed consent prior to the experiment and received monetary compensation for their participation. The experimental procedures adhered to the Declaration of Helsinki (2008) and were approved by the ethical committee of Sichuan Normal University.

#### 3.1.2 Stimuli and procedure

**Figure 3:**
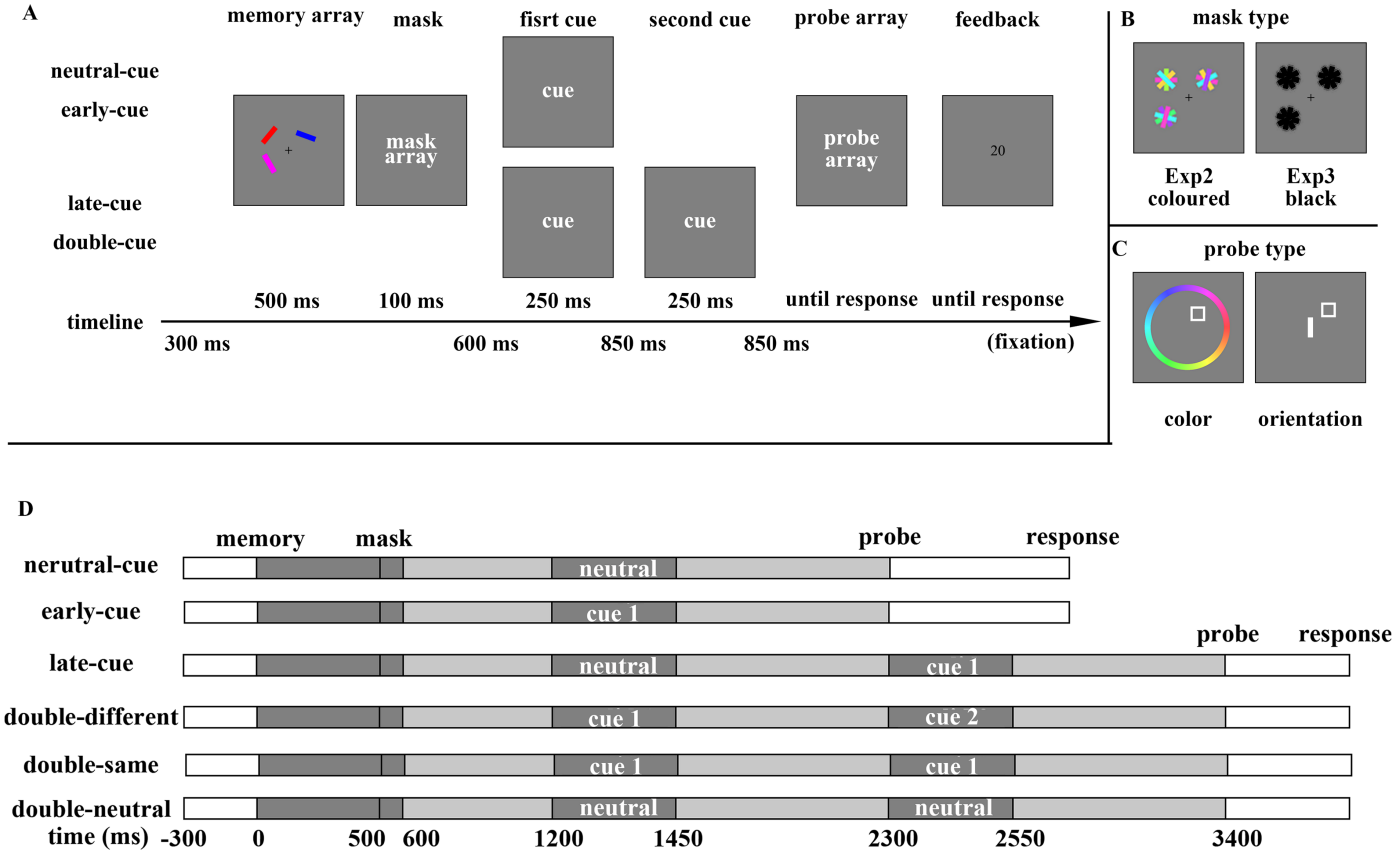
**(A)** The procedure for trials with one (upper) or two (lower) retro-cues in Experiment 2 and 3. **(B)** Mask array examples for Experiment 2 (left) and Experiment 3 (right). **(C)** Probe array examples of colors (left) and orientations (right). **(D)** The timeline of each cue type Experiments 2 and 3. Note that only four cue types (neutral-cue vs. early-cue vs. late-cue vs. double-different) were arranged in Experiment 2. Neutral cues showed the word “all.” “Cue 1” and “cue 2” showed the word “color” or “orientation.” Dark gray segments indicated the duration of cues. Different cues were numbered differently.

Visual masks were generated by randomly selecting a colored, oriented bar from the stimulus pool and intertwining it with three additional bars, ensuring that the orientations of the four bars were evenly spaced. The stimuli and apparatus used in Experiment 2 were identical to those in Experiment 1. The stimuli were selected from a pool of 360 continuous colors and 180 orientations (spanning the full 360° color space and 180° orientation range, respectively). In the memory array, each stimulus’s color and orientation differed by at least 60 color steps and 30° in orientation from any other stimulus, ensuring distinctiveness between items in the memory array. As shown in Figure 3, the main procedure began with a fixation cross presented for 300 ms, followed by a memory array displayed for 500 ms, featuring bars positioned at three of the four corners of an invisible square approximately 0.9° from the fixation point. Subsequently, a 100 ms display presented three masks at the same locations as the memory stimuli. The VWM retention period commenced with a 600 ms fixation interval, followed by one or two combinations of a cue array (lasting 250 ms) and an interval array (lasting 850 ms). Participants were instructed to trust and use the retro-cues, as extensive research has demonstrated their efficacy in improving memory performance. The probe array, which consisted of a white square indicating the probed item, followed. A colored wheel appeared during trials probing color, while a white bar appeared centered during trials probing orientation. Participants moved the mouse to select the appropriate level of the dimension being tested. The probe array remained on the screen until the participant clicked the mouse’s left button. Feedback, indicating the reproduction error (the absolute deviation between the reported and original levels of the target dimension in degrees), was provided at the end of each trial and remained until another mouse click. Emphasis was placed on accuracy rather than response speed.

Each cue type was presented across 100 trials probing color and 100 trials probing orientation, resulting in a total of 800 trials. The experimental factors, cue type (neutral-cue, early-cue, late-cue, and double-cue), and probe type (color vs. orientation) were randomly mixed. Participants completed 16 practice trials prior to the formal task. A rest break was provided every 10 min, with the entire duration of Experiment 2 being approximately 1 h.

#### 3.1.3 Data analysis

The errors for each participant and experimental condition were calculated by subtracting the value of the probed item from the corresponding response. To ensure the comparability of our findings with previous research on dimension-based RCB, we adopted the same dependent variable as used in previous studies (Liu et al., 2023; Ye et al., 2021; Niklaus et al., 2017; Ye et al., 2016; Hajonides et al., 2020). The primary dependent variable was the absolute deviation, referred to as the reproduction error. It is important to note that this offset is contingent upon the defined color step or orientation degree. Given the differences in response ranges between color (1–360 color steps) and orientation (1–180 orientation degrees), a larger reproduction error in color trials does not inherently indicate worse color memory performance compared to orientation memory performance. The qualitative differences in the color and orientation reports impelled us to conduct separate analyses for the reproduction error in the color and orientation report trials. To investigate the dimension-specific RCB and the use of the first cue, we compared reproduction errors between the single-cue conditions and the neutral-cue baseline. Additionally, to investigate the presence of unfocused dimension information in VWM indicated by the first cue, we compared the double-cue condition with the baseline. Two one-way repeated measures ANOVAs were conducted separately for color and orientation trials, with cue type (neutral-cue, early-cue, late-cue, double-cue) as the within-subject factor. The methods for estimating effect sizes and conducting follow-up pairwise comparisons were consistent with those used in Experiment 1. Moreover, the reproduction error data were analyzed using the mixture-swap model (Bays et al., 2009) through the MemToolbox (Suchow et al., 2013), with the results provided in Supplementary material. However, it is crucial to highlight that a few studies (van den Berg et al., 2014; van den Berg et al., 2012; Schurgin et al., 2020; Williams et al., 2023; Zhang and Lewis-Peacock, 2023; Williams et al., 2022) have advanced the models for VWM, which necessitate cautious interpretation of the results based on the mixture-swap model. Therefore, we primarily focused on the mean reproduction error as the key dependent variable to assess participants’ VWM performance.

### 3.2 Results

The results of the repeated measures ANOVA for color report trials revealed a significant main effect of cue type, *F*(3, 90) = 8.837, *p* < 0.001, η*_p_* 2 = 0.228. Meanwhile, the results of the ANOVA for orientation report trials revealed a similar pattern as those for color report trials, showing a significant main effect of cue type, *F*(3, 90) = 27.676, *p* < 0.001, η*_p_* 2 = 0.480.

#### 3.2.1 The dimension-based single cue benefit

**Figure 4:**
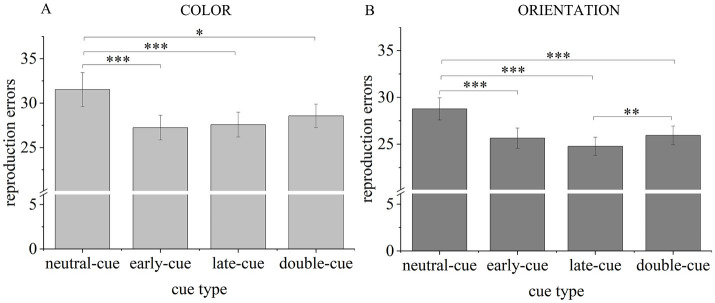
Reproduction error results of Experiment 2. A higher bar with larger reproduction errors reflects a worse performance in VWM task. Light gray bars represent mean reproduction errors for color report trials **(A)**. Dark gray bars represent mean reproduction errors for orientation report trials **(B)**. Error bars indicate SE. *p < 0.050, **p < 0.010, ***p < 0.001.

For color report trials (Figure 4A), planned comparisons revealed that participants’ reproduction errors were significantly lower in the single-cue trials than in the neutral-cue trials [early-cue vs. neutral-cue: *t*(30) = 3.987, *p* < 0.001, Cohen’s *d* = 0.716, BF_10_ = 74.777; late-cue vs. neutral-cue: *t*(30) = 3.759, *p* < 0.001, Cohen’s *d* = 0.675, BF_10_ = 43.019]. These results indicated the robust dimension-based RCB for color information.

Comparably, for orientation report trials (Figure 4B), planned comparisons revealed lower reproduction errors in the single-cue trials than in the baseline [early-cue vs. neutral-cue: *t*(30) = 6.547, *p* < 0.001, Cohen’s *d* = 1.176, BF_10_ > 1,000; late-cue vs. neutral-cue: *t*(30) = 10.159, *p* < 0.001, Cohen’s *d* = 1.825, BF_10_ > 1,000]. These results suggested the robust dimension-based RCB for orientation information.

Furthermore, participants’ performances for each dimension in single-cue conditions were independent from the onset of valid retro-cues [early-cue vs. late-cue, color: *t*(30) = 0.541, *p* = 0.593, Cohen’s *d* = 0.097, BF_01_ = 4.560; early-cue vs. late-cue, orientation: *t*(30) = 1.918, *p* = 0.065, Cohen’s *d* = 0.344, BF_01_ = 1.038].

#### 3.2.2 The dimension-based double cue benefit

Participants remembered better in trials with double cues than in trials with a neutral cue, regardless of the probed dimension [color: *t*(30) = 2.354, *p* = 0.025, Cohen’s *d* = 0.423, BF_10_ = 2.050; orientation: *t*(30) = 5.242, *p* < 0.001, Cohen’s *d* = 0.941, BF_10_ > 1,000]. These results indicated that the VWM performance was improved by attention guided by the second retro-cue.

#### 3.2.3 The comparison of double-cue and single-cue conditions

The performance for colors did not differ between valid cue types [early-cue vs. double-cue: *t*(30) = 2.029, *p* = 0.051, Cohen’s *d* = 0.364, BF_01_ = 0.866; late-cue vs. double-cue: *t*(30) = 1.394, *p* = 0.173, Cohen’s *d* = 0.250, BF_01_ = 2.173]. These results indicate that the improvement provided by the late color cue was comparable to that provided by the second color cue in the double-cue condition, indirectly suggesting that the unfocused color from the first cue display was not removed in the double-cue trials. In addition, there was a strong trend indicating that the improvement from the early color cue was more efficient than that from the second color cue.

However, when the orientation was probed, performances were worse in the double-cue condition than in the late-cue condition, *t*(30) = 2.907, *p* = 0.007, Cohen’s *d* = 0.522, BF_10_ = 6.187. This result suggests that the prior shift to another dimension may have influenced attention’s ability to prioritize orientation information in VWM; that is, the number of attention shifts impacted the prioritization effect for orientations. Performance did not differ between the double-cue and early-cue conditions, *t*(30) = 0.576, *p* = 0.569, Cohen’s *d* = 0.104, BF_01_ = 4.447, suggesting that the improvement provided by the early orientation cue was comparable to that of the second orientation cue.

### 3.3 Discussion

Experiment 2 demonstrated a robust dimension-based RCB across various conditions, including different probed dimensions, cue numbers, and cue onsets. This benefit was observed even under early-cue conditions, where participants were exposed to mixed cue types across trials. This suggests that participants likely allocated attention to a specific dimension upon the first cue display, leaving the probed dimension in double-cue trials unattended initially. Moreover, performance in double-cue trials did not deteriorate compared to neutral-cue trials. These findings collectively indicate that eliminating the unfocused dimension is not necessary for achieving the dimension-based RCB.

The consistency of the dimension-based RCB across color and orientation tasks in Experiment 2 suggests that a recall task might be more sensitive to detecting this effect than a change detection task, which aligns with our previous findings (Liu et al., 2023). As hypothesized in the discussion of Experiment 1, this discrepancy may be due to the distinct probe precision required by the recall task and change detection task. In Experiment 2, reproduction errors showed subtle differences in participants’ chosen colors or orientations, with changes as small as one color step or orientation degree. Conversely, in Experiment 1, the changes in probed colors and orientations were much larger, at least 60 color steps or 30 orientation degrees, respectively. As shown in Figure 4, the mean reproduction errors in Experiment 2 were smaller than the change thresholds used in Experiment 1 despite a higher memory load in Experiment 2. This suggests that subtle differences in orientation probes were captured more effectively by reproduction errors in the recall task than by accuracy in the change detection task. While the similarity between memory and probe stimuli can be increased in the change detection task, the probe precision of change detection tasks is inherently lower due to their binary nature, making it difficult to detect very small changes compared to the recall task.

To rule out the possibility that participants delayed processing the first cue until confirming the absence of a second cue, we included an early-cue condition in our task and compared its performance against the baseline condition. Although the color wheel or white bar in the probe array served as a valid cue, the decision-making process may have disrupted the resource reallocation. By including a blank interval between the retro-cue and probe array, we separated these processes. Ye et al. (2016) manipulated this interval and found that participants required more than 450 ms to effectively use a dimension-based retro-cue before the probe appeared. Thus, in our Experiment 2, the probe array appeared at the same time in early-cue trials as the second cue in late-cue or double-cue trials, preventing participants from using the post-cue interval to judge the cue type. Delaying the use of first cue would leave no time to use it before decision-making, thereby nullifying the dimension-based RCB in the early-cue condition. However, the better performance in early-cue trials compared to baseline rules out the possibility to use this strategy.

We also found no significant differences between performances in two single-cue conditions, particularly for probed colors, consistent with the findings of the study by van Moorselaar et al. (2015). One possible explanation for this result is that during the retention period (1,800 ms in our Experiment 2), the effect of focused attention on benefiting VWM may be independent of retro-cue onset. Previous research has demonstrated robust maintenance of representations for approximately 4 s (Zhang and Luck, 2009), indicating that the intrinsic properties of VWM representations do not differ at the onset of early or late cues, resulting in similar VWM performance once focused attention has been allocated.

Another possible explanation for these results is that memory representations may degrade when participants attempt to maintain all stimuli. Although participants in the early-cue condition could shift attention earlier to protect the cued dimension, participants in the late-cue condition might gain a unique advantage not present in the early-cue condition. Once familiar with the experimental design, participants would know that after the late cue, no additional cues would appear, and the last cue would always be 100% valid. At this point, they could strategically choose to remove the uncued dimension of the final cue from VWM, allowing more resources to protect the cued dimension. The advantage gained from this information removal in the late-cue condition may be comparable to the early prioritization/early detection advantage in the early-cue condition. This could explain why no performance differences were observed between the early-cue and late-cue conditions.

Interestingly, performance was better in late-cue trials than in double-cue trials when orientation was probed. The cue onsets were identical in both conditions. The only variation was the content of an early displayed cue. The color cue, in double-cue condition, elicited an accumulation of attention on color information in VWM. Previous studies found that participants required some time without disruption to deploy attention and use the retro-cue (Liu et al., 2023; Janczyk and Berryhill, 2014). Despite the comparable duration of attention guided by the first and second cues, our findings suggest that the benefit of refocusing attention on orientation information in the double-cue trials may have been less than the benefit of directly focusing attention on orientation information in the late-cue trials. In other words, the effectiveness of attention in benefiting VWM diminishes when attention shifts from one dimension to another. The number of attention shifts influences the dimension-based RCB, even when the duration of focused attention is sufficient for cue utilization.

An alternative explanation for the worse VWM performance in double-cue trials compared to late-cue trials may involve the unbalanced complexity of the masks used for each dimension. In Experiment 2, masks were generated from bars with 180 possible orientations but 360 possible colors, potentially leading to greater interference in color representations than in orientation ones. After an early cue directed attention to colors, VWM may have required more attention to differentiate between maintained color information and sensory inputs from the masks, thereby impairing the successive deployment of attention to orientations in VWM after the second cue. Performance for colors in single-cue trials, particularly in early-cue trials, tended to be better than in double-cue trials. This might indicate that participants prioritized distinguishing memory colors from mask colors, allocating more attention to color information in VWM. Near the beginning of the maintenance phase, an early color cue could facilitate this differentiation. However, an early orientation cue might demand more resources to reallocate attention than a color cue; meanwhile, the mask colors interfered with the unfocused memorized colors, possibly leading to worse performance in double-cue trials than in single-cue trials for color probes. Therefore, in Experiment 3, we used fixed colored, oriented masks to eliminate the influence of varying distraction levels across dimensions and to examine the impact of attention shift times on the dimension-based RCB.

Briefly, the results of Experiment 2 suggest that the dimension-based RCB does not require the removal of the unfocused dimension in VWM. Additionally, we infer that the prioritization of VWM by focused attention diminishes as the attention shift number increases in the dimension-based RCB. However, this inference faces some challenges in explaining the results for colors, likely due to different interference levels of masks on a different dimension.

## 4 Experiment 3: examining the influence of attention shift times for the dimension-based RCB

In Experiment 2, we did not observe a decline in VWM performance in the double-cue condition compared to the neutral-cue condition. Combined with the existence of dimension-based single-cue benefit, we hypothesize that the dimension probed in the double-cue condition remains in an unfocused state rather than being eliminated from VWM during the initial cue presentation. This suggests that VWM can benefit from dimension-based attention guided by a retro-cue without the complete removal of the unfocused dimension information. In addition, the gap of orientation performances between late-cue and double-cue trials in Experiment 2 reckoned that the shift times of attention might influence its improvement to orientation information in VWM. However, this effect was not observed in the color report trials. Thus, our current evidence supports that the initially unfocused dimension, when re-cued, is not removed from VWM and can be reinstated to a prioritized position. Nevertheless, it remains unclear whether the number of attentional shifts affects cue utilization.

For further investigation, we considered that the mask colors in Experiment 2 might have exerted stronger interference on representations than mask orientations and that the focused color information in VWM consumed substantial resources to resist this interference, thereby hindering subsequent attention shifts to orientation information. To address this, in Experiment 3, we used black masks with fixed orientations (0°, 45°, 90°, 135°) for each composing bar, similar to the study by van Moorselaar et al. (2014) to control the influence of masks on each memory dimension. By using masks with consistent features across trials, we aimed to reduce their salience, which could diminish their interference with VWM in turn.

Additionally, we included two additional cue conditions in Experiment 3: the double-neutral-cue condition, which presented two sequential neutral cues, and the double-same-cue condition, which presented two identical valid cues successively. The double-neutral-cue condition was comparable to other conditions with double cues not only on the total maintenance duration of representations but also on the number and appearance time of retro-cues. The semantic cues might be regarded as an outer signal to disrupt the existing attention allocation. Therefore, the double-neutral-cue condition may serve as a baseline for examining the impact of attentional shifts on the memory of dimensions. The double-same-cue condition was added to test the enhancement hypothesis of the dimension-based RCB. If participants performed better in double-same-cue trials compared to single-cue trials, we hypothesized that the focused dimension in VWM would receive enhancement from each retro-cue. Therefore, Experiment 3 included six cue conditions: neutral-cue, early-cue, late-cue, double-different-cue (same as the double-cue condition in Experiment 2), double-same-cue, and double-neutral-cue.

### 4.1 Materials and methods

#### 4.1.1 Participants

To achieve the same sample size as in Experiment 2, we recruited a new cohort of 39 college students, applying the same criteria as in Experiments 1 and 2. Eight students were excluded due to a floor effect in their performance for one condition (reproduction errors >70), resulting in a final sample of 31 participants (one left-handed, 27 female participants, four male participants, mean age = 21.87 years, SD = 3.10) for further analysis. Our sample size is sufficient to detect a large effect size (η*_p_* 2 = 0.26) with 98% statistical power at a significance level of 0.05, as determined by a power analysis (Faul et al., 2007) for a one-way (6 levels) repeated measures ANOVA design. All participants provided informed consent prior to the experiment and received monetary compensation for their participation. The experimental procedures adhered to the Declaration of Helsinki (2008) and were approved by the ethical committee of Sichuan Normal University.

#### 4.1.2 Stimuli and procedure

The stimuli and apparatus in Experiment 3 were identical to those used in Experiment 2. The procedures in Experiment 3 also mirrored those in Experiment 2, with the exception of two additional cue types: in the double-same-cue trials, two sequential valid cues were directed at the same dimension; in the double-neutral-cue trials, neutral cues were presented twice in succession.

Each cue type was presented across 48 trials for each probed dimension, resulting in a total of 576 trials. The experimental factors of cue type (neutral-cue vs. early-cue vs. late-cue vs. double-different-cue vs. double-same-cue vs. double-neutral-cue) and probe type (color vs. orientation) were randomly mixed. Participants completed 24 practice trials before beginning the main experiment. A rest break was provided every 10 min, with the entire experiment lasting approximately 90 min.

#### 4.1.3 Data analysis

The data analysis for Experiment 3 was conducted as described for Experiment 2, except that ANOVAs were conducted with six cue types. In addition, we combined the data for the same four cue types (neutral-cue, early-cue, late-cue, double-cue) in Experiments 2 and 3, forming a large sample of 62 (two left-handed, 54 female participants, eight male participants, mean age = 21.15 years, SD = ± 2.53) to examine the stability of the single- and double- double-based RCB. Data analyses for this large sample were identical to analyses in Experiment 2. Furthermore, due to the relatively low number of trials per condition in Experiment 3, we did not conduct additional model fitting using the swap model, as it typically requires a larger number of trials for reliable fitting. However, we have uploaded the raw trial-by-trial data for each participant to the Open Science Framework at https://osf.io/vmsqa/ to allow other researchers to further analyze our data.

### 4.2 Results for experiment 3

The results of the repeated measures ANOVA revealed a significant main effect of cue type, for color report trials, *F*(5, 150) = 7.759, *p* < 0.001, η*_p_* 2 = 0.205, rather than for orientation report trials, *F*(5, 150) = 2.044, *p* = 0.076, η*_p_* 2 = 0.064.

Planned comparisons revealed no significant difference of reproduction errors between neutral-cue and double-neutral-cue trials, regardless of the probed dimension [color: *t*(30) = 0.529, *p* = 0.601, Cohen’s *d* = 0.095, BF_01_ = 4.585; orientation: *t*(30) = 0.539, *p* = 0.594, Cohen’s *d* = 0.097, BF_01_ = 4.564]. Therefore, the VWM performance for each dimension was independent of the maintenance duration when only neutral cues appeared.

#### 4.2.1 The dimension-based single cue benefit

**Figure 5:**
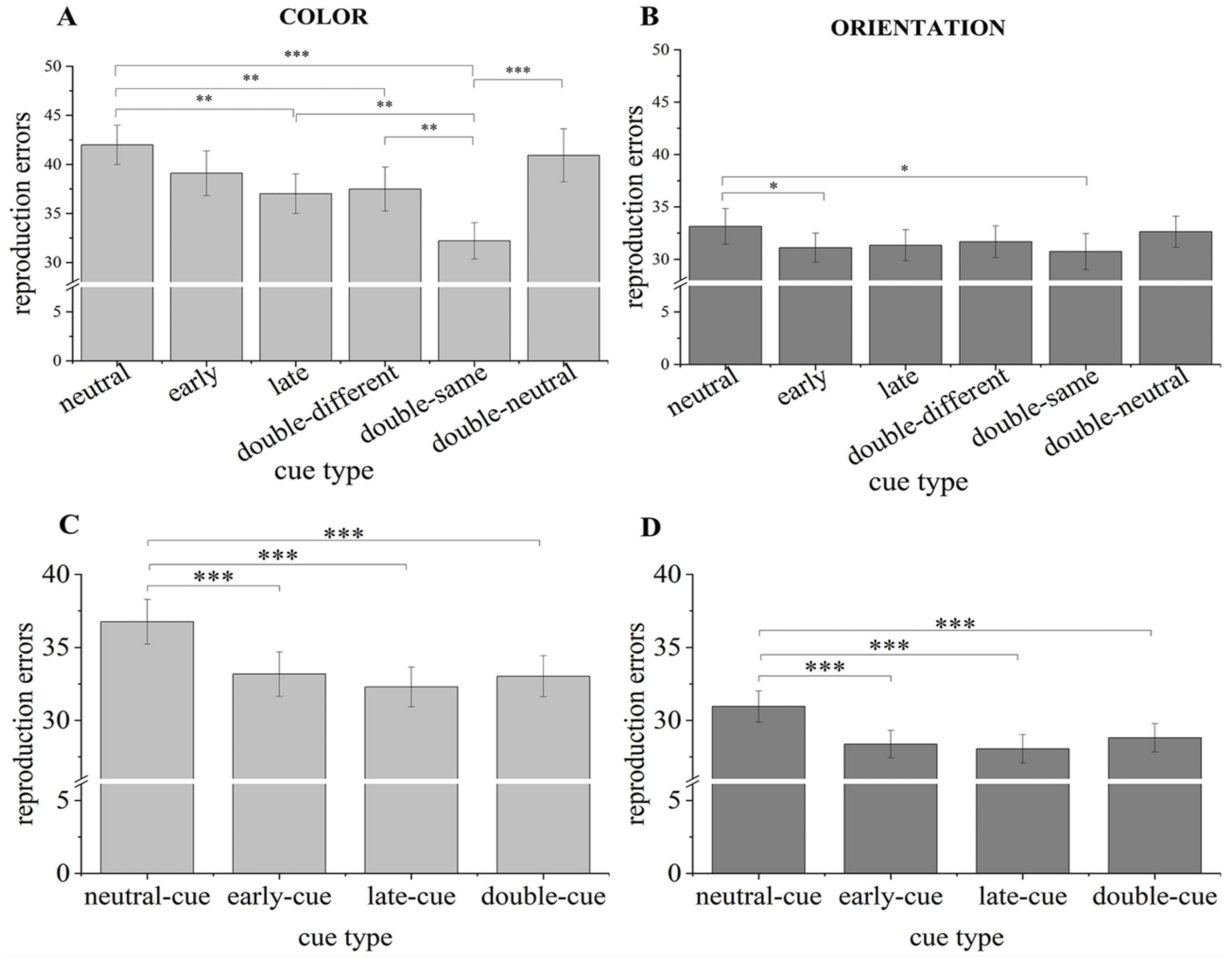
Reproduction error results of Experiment 3 and the compound data from Experiments 2 and 3. Light gray bars represent mean reproduction errors for color report trials in Experiment 3 **(A)**. Dark gray bars represent mean reproduction errors for orientation report trials in Experiment 3 **(B)**. Light gray bars represent mean reproduction errors for compound data of color report trials in Experiments 2 and 3 **(C)**. Dark gray bars represent mean reproduction errors for compound data of orientation report trials in Experiments 2 and 3 **(D)**. Error bars indicate SE. *p < 0.050, **p < 0.010, ***p < 0.001.

Participants’ reproduction errors for color reports (Figure 5A) were significantly lower in the late-cue trials than in the neutral-cue trials, *t*(30) = 3.174, *p* = 0.003, Cohen’s *d* = 0.570, BF_10_ = 11.072. This finding suggests that the late color retro-cue provided a notable benefit for the color information stored in VWM. However, we did not find strong evidence to support better performance in the early-cue condition than in the neutral-cue condition, *t*(30) = 1.837, *p* = 0.076, Cohen’s *d* = 0.330, BF_01_ = 1.177. This result implies that the advantage gained from the early color retro-cue for color information was relatively weak, or that participants may not have efficiently utilized the initial color cue.

Aligned with orientation results in Experiment 2, participants’ reproduction errors (Figure 5B) were significantly lower in the early-cue trials than in the neutral-cue condition, *t*(30) = 2.338, *p* = 0.026, Cohen’s *d* = 0.420, BF_10_ = 1.991. However, results proved a trend of better performances in the late-cue trials than in the neutral-cue condition, *t*(30) = 1.955, *p* = 0.060, Cohen’s *d* = 0.351, BF_01_ = 0.977. These results indicate that the use of an orientation single-cue provided a benefit for the corresponding information stored in VWM. Furthermore, participants’ performances for each dimension in single-cue conditions were independent from the onset of valid retro-cues [early-cue vs. late-cue, color: *t*(30) = 1.465, *p* = 0.153, Cohen’s *d* = 0.263, BF_01_ = 1.988; early-cue vs. late-cue, orientation: *t*(30) = 0.261, *p* = 0.796, Cohen’s *d* = 0.047, BF_01_ = 5.058].

#### 4.2.2 The dimension-based double cue benefit

Participants remembered colors better in trials with double different cues than in trials with a neutral cue, *t*(30) = 3.221, *p* = 0.003, Cohen’s *d* = 0.579, BF_10_ = 12.299. This result suggested that attention guided by the second color cue enhanced the corresponding memory. However, this significance disappeared when they remembered orientations, *t*(30) = 1.463, *p* = 0.154, Cohen’s *d* = 0.263, BF_01_ = 1.995. This result provided evidence for no worse performance for orientation in double-cue trials than in neutral-cue trials.

In addition, we compared the performance for the double-different-cue and the double-neutral-cue conditions but found no robust significant differences [color: *t*(30) = 1.476, *p* = 0.150, Cohen’s *d* = 0.265, BF_01_ = 1.961; orientation: *t*(30) = 1.072, *p* = 0.292, Cohen’s *d* = 0.193, BF_01_ = 3.089]. Although neutral cues contained no information for the to-be-probed dimension, they appeared in the visual field and might activate participants during the boring maintenance phase. The overall activation might benefit each dimension memory, causing no observable differences between performance for the double-different-cue and double-neutral-cue trials.

#### 4.2.3 The comparison of double-cue and single-cue conditions

In color report trials, the performance did not differ between the double-different-cue and single-cue conditions [early-cue vs. double-different-cue: *t*(30) = 0.954, *p* = 0.348, Cohen’s *d* = 0.171, BF_01_ = 3.440; late-cue vs. double-different-cue: *t*(30) = 0.307, *p* = 0.761, Cohen’s *d* = 0.055, BF_01_ = 4.997]. This result indicated that the attention shift times did not influence attention enhancement on color information in VWM.

Similarly, in orientation report trials, the performance did not differ between the double-different-cue and single-cue conditions [early-cue vs. double-different-cue: *t*(30) = 0.807, *p* = 0.426, Cohen’s *d* = 0.145, BF_01_ = 3.867; late-cue vs. double-different-cue: *t*(30) = 0.372, *p* = 0.712, Cohen’s *d* = 0.067, BF_01_ = 4.895]. This result suggested that the attention shift times did not influence attention enhancement on orientation information in VWM.

Interestingly, across comparisons between conditions in which the late displayed color cue were valid, participants performed better in the double-same-cue condition than in other conditions [double-same-cue vs. late-cue: *t*(30) = 3.235, *p* = 0.003, Cohen’s *d* = 0.581, BF_10_ = 12.674; double-same-cue vs. double-different-cue: *t*(30) = 3.041, *p* = 0.005, Cohen’s *d* = 0.546, BF_10_ = 8.247]. This result indicated that color information in VWM may benefit from each time of attention focus. However, this significant difference disappeared when orientations were probed [double-same-cue vs. late-cue: *t*(30) = 0.624, *p* = 0.537, Cohen’s *d* = 0.112, BF_01_ = 4.360; double-same-cue vs. double-different-cue: *t*(30) = 1.013, *p* = 0.319, Cohen’s *d* = 0.182, BF_01_ = 3.265]. This result suggested that orientation information in VWM only got limited benefit from focused attention regardless of attention focus times.

### 4.3 Results for the compound sample of experiments 2 and 3

With a larger sample, results of the repeated measures ANOVA revealed a significant main effect of cue type regardless of probed dimension [color: *F*(3, 183) = 9.920, *p* < 0.001, η*_p_* 2 = 0.140; orientation: *F*(3, 183) = 13.531, *p* < 0.001, η*_p_* 2 = 0.182].

#### 4.3.1 The dimension-based single-cue benefit

Participants performed better in the single-cue conditions than in the neutral-cue condition [for color report (Figure 5C), early-cue vs. neutral-cue: *t*(61) = 3.782, *p* < 0.001, Cohen’s *d* = 0.480, BF_10_ = 67.319; late-cue vs. neutral-cue: *t*(61) = 4.756, *p* < 0.001, Cohen’s *d* = 0.604, BF_10_ > 1,000; for orientation report (Figure 5D), early-cue vs. neutral-cue: *t*(61) = 5.196, *p* < 0.001, Cohen’s *d* = 0.660, BF_10_ > 1,000; late-cue vs. neutral-cue: *t*(61) = 5.620, *p* < 0.001, Cohen’s *d* = 0.714, BF_10_ > 1,000]. These results indicated the dimension-based RCB for each dimension.

Furthermore, participants’ performances for each dimension in single-cue conditions were independent from the onset of valid retro-cues [early-cue vs. late-cue, color: *t*(61) = 1.111, *p* = 0.271, Cohen’s *d* = 0.141, BF_01_ = 3.998; orientation: *t*(61) = 0.642, *p* = 0.523, Cohen’s *d* = 0.082, BF_01_ = 5.903].

#### 4.3.2 The dimension-based double-cue benefit

Participants also remembered better in trials with double cues than in trials with a neutral cue [color: *t*(61) = 3.980, *p* < 0.001, Cohen’s *d* = 0.505, BF_10_ = 122.015; orientation: *t*(61) = 3.768, *p* < 0.001, Cohen’s *d* = 0.479, BF_10_ = 64.695]. These results suggest that VWM for each dimension benefited from the addition of a second retro-cue.

#### 4.3.3 The comparison of double-cue and single-cue conditions

The performance did not differ between the double-cue and single-cue conditions, both in color report trials [early-cue vs. double-cue: *t*(61) = 0.162, *p* = 0.871, Cohen’s *d* = 0.021, BF_01_ = 7.099; late-cue vs. double-cue: *t*(61) = 0.861, *p* = 0.393, Cohen’s *d* = 0.109, BF_01_ = 5.049], and in orientation report trials [early-cue vs. double-cue: *t*(61) = 0.999, *p* = 0.322, Cohen’s *d* = 0.127, BF_01_ = 4.469; late-cue vs. double-cue: *t*(61) = 1.517, *p* = 0.135, Cohen’s *d* = 0.193, BF_01_ = 2.432]. These results suggest that the number of the attention shifts may not significantly influence the benefits gained by VWM from focused dimension-based attention.

### 4.4 Discussion

In Experiment 3, we used masks with fixed features to control their interference on each dimension of the representations, aiming to investigate whether the attention shift times influenced the prioritization of dimension information in VWM. We found no significant difference in VWM performance between the double-different-cue and late-cue conditions. Given that the dimension-based single cue benefit for orientations was evident in the early-cue condition, and that the comparison showed better memory performance for colors in the early-cue trials compared to the neutral-cue trials, we inferred that the probed dimension in the double-different-cue trials was stored in an unfocused state during the first cue display.

To further confirm this, we combined data from Experiments 2 and 3, which had comparable sample sizes and procedures, across four cue type conditions (neutral-cue, early-cue, late-cue, double-different-cue). The comparisons between participants’ performances in double-different-cue and single-cue trials revealed no significant differences. This suggests that the number of attention shift between dimensions may not influence the benefits gained by VWM from focused dimension-based attention.

Consistent with the results from Experiment 2, we did not observe worse performance in the double-different-cue condition compared to the neutral-cue or double-neutral-cue conditions, which does not support the necessity of forgetting unfocused dimensions to achieve the dimension-based RCB. Furthermore, a robust dimension-based double cue benefit for both color and orientation was found only through the combined data analysis with a larger sample size. This evidence further supports that dropping the contents of one dimension from VWM is not necessary for dimension-based RCBs to emerge.

Interestingly, we observed differences in the improvement of VWM by attention across dimensions. In the double-same-cue condition, VWM for colors was the best among all color-reporting trials with double cues. This finding suggested that color representations were improved by a repetitive valid retro-cue, in alignment with the study by Rerko and Oberauer (2013), who observed that VWM performances of colors in trials with successive three retro-cues (orienting to object 1, object 2 and object 1 respectively) were better than those in trials with single retro-cue.

The advantage of the double-same-cue condition may be due to the participants gaining incremental benefits from each retro-cue. When the first cue appeared, participants could shift their attention to the color dimension, prioritizing color information while the orientation dimension remained in VWM as unprioritized information. When the second retro-cue appeared, participants were aware that the upcoming report would focus on the color dimension, allowing them to discard the unfocused orientation information from VWM and allocate more resources to protect the prioritized color information. Therefore, in the double-same-cue condition, participants had more opportunities to gain benefits from the retro-cue for the probed dimension compared to the single-cue and double-different-cue conditions.

This effect was especially pronounced when the cued dimension was color. Although we also observed an advantage in the double-same-cue condition over the early-cue condition when the cued dimension was an orientation, the improvement was not as pronounced as when the cued dimension was color. One possible explanation is that, in the color double-same-cue condition, participants could use the second cue to discard the orientation information from VWM, thereby freeing up significant VWM resources for color. In contrast, in the orientation double-same-cue condition, participants could only use the second cue to discard the color information, which, as previous research has shown, occupies less VWM capacity than orientation information (Miller et al., 2014; Hao et al., 2018). Thus, the amount of free VWM resources gained by discarding color information was more limited, resulting in a less pronounced advantage for the orientation double-same-cue condition compared to the color condition.

Experiments 2 and 3 in the present study showed consistent performance between two single-cue conditions, which proved similarity as previous results of object-based retro-cues (van Moorselaar et al., 2015) but contradicts those observed by Rerko and Oberauer (2013). These complex results might be explained by differences in temporal manipulations and memory load across studies. van Moorselaar et al. (2015) used a memory-to-late-cue interstimulus interval (ISI) of 1,100 ms, providing a stable maintenance duration for VWM representations in their study. Rerko and Oberauer (2013) used a relatively longer ISI of 1,600 ms, during which VWM performance declined. In our study, the memory-to-late-cue ISI was even longer, at 1,800 ms, which theoretically could lead to a decay in representations before the late cue display, assuming memory load differences were disregarded. However, we presented three bars in each trial for memorizing, which is a smaller memory load compared to previous studies (8 orientations, van Moorselaar et al., 2015; 6 colors, Rerko and Oberauer, 2013). Maintaining fewer items might be easier for limited VWM capacity, potentially leading to a longer duration without significant decay (Vogel et al., 2001). The lack of significant differences between single- and double-neutral-cue conditions in present study also support robust maintenance of VWM representations for 2,900 ms (the memory-to-probe ISI in double-neutral-cue trials).

It is important to note that while we have highlighted some similarities and contradictions between our findings of the dimension-based RCB in the current study and previous findings on the object-based RCB, these benefits likely arise from fundamentally different mechanisms due to the integrated nature of VWM representation storage. Previous studies have also acknowledged that dimension-based attention influences VWM maintenance differently than object-based attention (Liu et al., 2023; Hajonides et al., 2020; Lin and Luck, 2009).

## 5 General discussion

In the current study, we manipulated the number of dimension-based retro-cues in VWM tasks with varying probe precisions (a change detection task in Experiment 1; a recall task in Experiments 2 and 3) to investigate whether VWM requires the removal of the unfocused dimension to achieve the dimension-based RCB. Across all three experiments, we consistently observed a single-cue benefit in dimension-probed VWM tasks, aligning with previous research on dimension-based retro-cues (Liu et al., 2023; Niklaus et al., 2017; Park et al., 2017; Ye et al., 2016). Due to the mixed arrangement of cue types, participants were unable to distinguish between early-cue and double-cue conditions when they saw the early displayed cue. This consistent finding suggests that the probed dimension in double-cue trials was unfocused during the first cue array. Consequently, we infer that the unfocused dimension was not removed from VWM during the dimension-based single-cue benefit, as we did not observe worse performance in double-cue trials compared to the no/neutral-cue baseline.

### 5.1 Cognitive mechanisms of dimension-based RCB

The dimension-based RCB has been explained by a dual mechanism involving both prioritizing the focused dimension and removing the unfocused dimension from VWM (Ye et al., 2016). When participants store stimuli with multidimensional features, the prolonged retention time and competition between different dimensions may cause the information maintained in VWM to decay. However, when a retro-cue is presented, participants can shift their attention to a specific dimension, enhancing its priority and protecting it from decay, or they may choose to forget the unfocused dimension. This reduces competition between dimensions and frees up memory resources for redistribution to the target dimension. However, the current study suggests that the removal of the unfocused dimension is not a necessary condition for the dimension-based RCB, although we do not dispute the validity of the removal mechanism. Our results indicate that the removal mechanism is not essential for the protection mechanism to occur. The prioritization mechanism alone can facilitate the dimension-based RCB without the involvement of the removal process. Liu et al. (2023) inserted sensory masks after the retro-cue to examine the requirement of sustained attention for dimension-based RCB. Their results showed that the dimension-based RCB effect existed when the mask appeared after 1,400 ms from the onset of a retro-cue, but the effect was weaker compared to conditions with no masks. Therefore, the sustained attention facilitates the effective usage of dimension-based retro-cues but is not necessary. In trials with masks, the enhancement of focused dimension was ineffective because of the limited time without disruption, but VWM removed information of the unfocused dimension fast. Thus, in the previous study (Liu et al., 2023), the removal mechanism played a critical role in explaining the dimension-based RCB under masked conditions. However, in the current study, the unfocused dimension in the first cue display might be probed, encouraging participants not to remove it from VWM. Taken together, these findings suggest that participants flexibly use dimension-based retro-cues, choosing whether or not to remove unfocused dimensional information from VWM while prioritizing the target dimension, depending on the task requirements.

Previous studies have also suggested for the prioritization account and the removing account underlying the object-based RCB (van Moorselaar et al., 2015; Astle et al., 2012; Fu et al., 2022; Günseli et al., 2015; Heuer and Schubo, 2016; Kuo et al., 2012; Rerko et al., 2014; Souza and Oberauer, 2016; Souza et al., 2016). To discern the differences in mechanisms between dimension-based and object-based RCBs, it is essential to first define the nature of dimension storage in VWM. Ngiam (2024) proposed a memory for latent representations (MRL) model to reconcile controversial accounts of VWM storage. According to the MLR model, dimension inputs are bound with contextual information and stored in a binding pool, which aligns with findings that dimensions can be stored and forgotten independently (Fougnie and Alvarez, 2011; Markov et al., 2019). Meanwhile, some integrated tokens based on context correspond to cell assemblies that represent dimensions in binding pool. Signals for a token are effective to activate the whole cell assembly, that is, the dimensions bound to the same object, which explains the integration effect based on objects (Fougnie et al., 2013). When we use the VWM in tasks, the dimension information stored in the binding pool might be directly activated; alternatively, the token on target location is identified, activating the dimension information. The choice between activating the dimension or the token depends on the task requirements.

In terms of the object-based RCB, the token of the unfocused location might be removed to free up VWM resources for target storage. According to the prioritization account, the retro-cue activates the target tokens and guides the decoding of corresponding information into a variational autoencoder, an online processing platform of VWM, for prioritization. However, pertaining to the dimension-based RCB, dimension information in the binding pool is directly decoded to variational autoencoder or removed, independent from the process of tokens. Therefore, although part of information might be removed from VWM after a dimension-based retro-cue, the number of tokens does not change. The removal reduces competition among information in the binding pool but does not decrease the memory load. This might explain the weak dimension-based RCB effect, which cannot be observed sometimes with low-precision change detection tasks but the object-based one is strong (Hajonides et al., 2020; Lin et al., 2021). Additionally, in our experiments, participants did not remove information from the unfocused dimension when the early cue was presented. They had to use limited VWM resources to prioritize the decoded information while simultaneously maintaining the unfocused information. Therefore, the dimension-based RCB was weak. The maintained unfocused information might suffer from internal competition due to limited VWM resources, leading to a trend of worse VWM performance for refocused orientations in double-different-cue trials compared to focused orientations in late-cue trials.

For the underlying neural mechanism, an object’s representation in VWM is maintained by a synchronously firing cell assembly, with different groups of neurons coding for different dimensions (Luck and Vogel, 2013). In the recurrent feedback loop model, only one cell assembly fires at a given moment. A cell assembly gains from each firing and decays in the interval not firing. A representation that remains inactive for too long will be lost. With the guidance of object-based retro-cue, the cell assembly representing target object might fire for longer time. Correspondingly, unfiring intervals for other representations is prolonged, increasing the abandonment risk of unfocused object representations. For the effect of dimension-based attention, however, the section coding a focused dimension in each cell assembly is synchronized with that of unfocused dimension. The prioritization of attention based on dimension may be achieved by having more neurons represent the focused dimension, but during the maintenance phase, shifting the attentional focus is unlikely to influence the frequency with which each cell assembly fires. As a result, the unfocused dimension in VWM may not be removed.

### 5.2 Removal mechanism in the double-cue condition

The results from our three experiments consistently demonstrate that VWM performance for refocused dimensions in double-(different-)cue trials showed benefits compared to neutral/no-cue trials and did not differ from performance for focused dimensions in single-cue trials, regardless of the dimension type or single-cue onset timing. However, we do not think this suggests that uncued dimensions are immune to fidelity costs.

Previous research has shown that representations maintained over the short term can also be stored in a passive state, without persistent neural activity (Larocque et al., 2014; Rose et al., 2016). In the passive state, short-term maintenance might be achieved through weight-based changes in synaptic connectivity, which are not detectable using standard recording methods (Stokes, 2015; Wolff et al., 2017). When information is not immediately needed, it may be transferred into this passive state until it is reactivated into an active state. In our double-different-cue trials, when the first cue appeared, participants could have transferred information about the unfocused dimension into this passive state (Li et al., 2020). However, previous research has shown that although representations stored in the passive state are robust with little memory loss during latent retention, they are susceptible to impairment during storage state switching (Zhang et al., 2022). Furthermore, our recent study suggests that, unlike object-based RCB, sustained attention is required to achieve effective dimension-based RCB (Liu et al., 2023). Thus, when the first cue appears, unfocused dimension information is not completely forgotten, but its maintenance may still be impaired due to attentional shifts and storage state switching. This aligns with previous dimension-based RCB studies (Niklaus et al., 2017; Park et al., 2017), which have found significant retro-cue costs in invalid cue trials.

In our study, we did not observe differences in memory performance for the dimension indicated by the second cue (i.e., the initially unfocused dimension) in double-different-cue trials compared to the RCB observed in single-cue trials. A reasonable explanation for this result is that, in double-different-cue trials, when the second cue appeared, participants not only shifted attention to the previously unfocused dimension, reactivating it from the passive state to the active state but also removed the initially focused dimension (indicated by the first cue) from VWM. This removal mechanism provided a unique benefit that compensated for the earlier impairment of the unfocused dimension, resulting in a similar level of dimension-based RCB in double-cue trials as in single-cue trials.

This removal mechanism was further supported by the results of Experiment 3 in the double-same-cue trials, where memory performance was better than in both single-cue and double-different-cue trials. In double-same-cue trials, the focused dimension not only avoided the costs associated with attentional shifts and storage state switching but also benefited from the removal of the uncued dimension when the cue was presented a second time, yielding an additional advantage.

Thus, while our study shows that individuals do not necessarily need to remove unfocused dimensions from VWM to achieve dimension-based RCB, our results also suggest that individuals can gain additional dimension-based RCB by removing unfocused dimensions from VWM. This removal mechanism can operate alongside the prioritization mechanism, with the decision to remove unfocused dimensions depending on task demands.

### 5.3 Limitations

Our study has some limitations. First, the actual validity of early- and late-displayed cues across all trials may have influenced participants’ expectations and response tendencies, complicating the attention focus trajectory in the current study. Although we attempted to mitigate this confounding variable by increasing the reliability of the early-displayed cue (actual validity of each cue = validity trials/appearance trials: Experiments 1 and 2: early-displayed cue = 50%, late-displayed cue = 100%; Experiment 3: early-displayed cue = 66.7%, late-displayed cue = 100%), this difference cannot be fully eliminated without the addition of an invalid second-cue condition. Future studies should explore the impact of expectations using invalid retro-cues. Second, the primary aim of this research was to assess the necessity of removing unfocused dimensions in achieving the dimension-based RCB. Consequently, we did not extensively investigate the prioritization account. Our results in Experiment 3 suggest potential differences in the prioritization of focused attention on colors versus orientations in VWM. Further studies could explore these differences using triple retro-cues or investigate the dimension specificity of attention enhancement across other dimensions, such as shapes, within the context of the dimension-based RCB. Third, in line with previous dimension-based RCB studies (Liu et al., 2023; Ye et al., 2021; Niklaus et al., 2017; Ye et al., 2016; Hajonides et al., 2020), we used absolute error as the main dependent variable in Experiment 2 and Experiment 3. This choice allowed for greater comparability with previous research but does not imply that we consider absolute error as the most sensitive measure of condition differences. In Supplementary material, we also conducted model fitting using the mixture-swap model on Experiment 2 data, as done in previous dimension-based RCB studies (Ye et al., 2016; Hajonides et al., 2020). This decision was similarly made for comparability purposes. However, we do not endorse the mixture-swap model as the optimal model for comparing condition differences. For example, recent studies suggest that the TCC (target confusability competition)-swap model may provide superior parameter estimates of the swap rate compared to the mixture-swap model (Williams et al., 2023; Williams et al., 2022). However, the choice of model can lead to different quantitative and qualitative interpretations of the data. Therefore, we caution against over-interpreting the model-fitting results in Supplementary material and chose not to focus extensively on these results in the main text. While our absolute error findings effectively address the central question of whether unfocused dimensions are forgotten, future researchers could apply models that offer superior parameter estimation to further explore other mechanisms underlying dimension-based RCB. Researchers are also encouraged to analyze our shared raw trial-by-trial data, available on the Open Science Framework at https://osf.io/vmsqa/, to verify their conclusions.

## 6 Conclusion

By manipulating the number of retro-cues in VWM tasks, we found that the removal of unfocused dimensions from VWM is not necessary for the dimension-based RCB effect. Additionally, the number of shifts in dimension-based attention does not affect the benefit derived from prioritizing specific dimensions. Our study provides new evidence on the mechanisms underlying the dimension-based RCB and offers insights into the storage and processing nature of VWM for multidimensional objects.

## Data Availability

The datasets presented in this study can be found in online repositories. The names of the repository/repositories and accession number(s) can be found at: https://osf.io/vmsqa/.
