# Supplementary material for: Dimension-based retro-cue benefit in working memory does not require unfocused dimension removal
Source: Front Psychol. 2024 Nov 1;15:1433405. doi: 10.3389/fpsyg.2024.1433405 (PMC11566143; doi:10.3389/fpsyg.2024.1433405)
Supplement: Supplementary file 1 [file Data_Sheet_1.PDF]

Dimension-Based Retro-Cue Benefit in Working Memory Does Not Require Unfocused Dimension Removal

Ruyi Liu [1,2,3], Lijing Guo [1,3], Xiaoshu Lin [3], Dan Nie [3], Piia Astikainen [3], Chaoxiong Ye [1,2,3,4*]

[1] School of Education, Anyang Normal University, Anyang, China;

[2] Institute of Brain and Psychological Sciences, Sichuan Normal University, Chengdu, China;

[3] Department of Psychology, University of Jyvaskyla, Jyväskylä, Finland;

[4] Faculty of medicine, University of Helsinki, Helsinki, Finland;

\+ Ruyi Liu and Lijing Guo contributed equally to this work and should be considered as co-first authors.

*** Correspondence:**
Chaoxiong Ye
cxye1988@163.com

The swap model parameter results of Experiment 2

We fitted the data in Experiment 2 using the swap model (Bays et al., 2009) with the MemToolbox (Suchow et al., 2013). The swap model posits that participants' behavior arises from a mixture of three trial types: first, trials in which participants successfully consolidated items into VWM, leading to a noisy representation of the target color or orientation, which follows a von Mises distribution; second, trials where participants failed to consolidate the items into VWM, resulting in random guessing of the reported color or orientation and yielding a uniform distribution; and third, trials where participants reported the non-target color or orientation during the response phase, producing a von Mises distribution centered around the non-target. This model enabled us to estimate the guess rate, the precision of memory representations (SD), and the non-target report rate.

Data Analysis

Data from eight participants were excluded from the analysis due to unsuccessful model fitting. To investigate the specific components of VWM performance affected by different types of dimension-based retro-cues, we examined guess rate, SD, and non-target report rate as indices of recall task performance. These dependent variables were analyzed separately for color and orientation report trials using one-way repeated measures ANOVAs, with cue type (neutral, early, late, double) as a within-subject factor. Separate repeated measures ANOVAs were conducted for each variable in both color and orientation report trials. Where significant main effects were observed, pairwise comparisons were employed for further exploration. Additionally, paired-samples t-tests and Bayes factor analyses were conducted to assess differences in cue efficiency. All tests were performed with a significance threshold set at $p < 0.05$. Effect sizes were estimated using partial eta-squared (η_p^2) for ANOVAs and Cohen's d for t-tests. Bayes factor (BF_{10}) analyses were included to determine the support for either the alternative or null hypothesis in the t-test results.

Results

Guess rate

The average guess rate for the color report trials and the orientation report trials are illustrated in SFigure 1A and SFigure 1B, respectively.

Results of the repeated measures ANOVA for color report trials revealed no significant main effect of cue type, $F(3, 66) = 0.661$, $p = 0.579$, $\eta_p^2 = 0.029$. Meanwhile, results of the ANOVA for orientation report trials revealed a similar

pattern as those for color report trials, showing no significant main effect of cue type, F (3,69) = 0.836, p = 0.479, η_p^2= 0.037.

The dimension-based single retro-cue benefit

For color report trials, planned comparisons revealed that participants' guess rate were no significant difference between the single-cue trials and the baseline (early-cue vs. neutral-cue: t (22) = 1.157, *p* = 0.260, Cohen's d = 0.241, BF_{10} = 0.396; late-cue vs. neutral-cue: t (22) = 0.674, *p* = 0.508, Cohen's d = 0.140, BF_{10} =0.269).

Comparably, for orientation report trials, planned comparisons revealed no difference between the single-cue trials and the baseline (early-cue vs. neutral-cue: t (22) = 0.098, *p* = 0.923, Cohen's d = 0.020, BF_{10} = 0.220; late-cue vs. neutral-cue: t (22) = 1.060, *p* = 0.301, Cohen's d = 0.221, BF_{10} = 0.361).

Furthermore, participants' guess rate for each dimension in single-cue conditions were independent from the onset of valid retro-cues (early-cue vs. late-cue, color: t (22) = 0.849, *p* = 0.405, Cohen's d = 0.177, BF_{10} = 0.302; early-cue vs. late-cue, orientation: t (22) = 1.021, *p* = 0.318, Cohen's d = 0.213, BF_{10} = 0.348).

The dimension-based double retro-cue benefit

Participants remember performance didn't show significantly difference between in trials with double cues and in trials with a neutral cue, regardless of the probed dimension (color: t (22) = 0.838, *p* = 0.411, Cohen's d = 0.175, BF_{10} = 0.300; orientation: t (22) = 0.336, *p* = 0.740, Cohen's d = 0.070, BF_{10} = 0.230).

The comparison of double-cue and single-cue conditions

The performance for colors did not differ between valid cue types (early-cue vs. double-cue: t (22) = 0.446, *p* = 0.660, Cohen's d = 0.093, BF10 = 0.239; late-cue vs. double-cue: t (22) = 0.282, *p* = 0.780, Cohen's d = 0.059, BF10 = 0.227).

Meanwhile, the performance for orientations did not differ between valid cue types (early-cue vs. double-cue: t (22) = 0.243, *p* = 0.811, Cohen's d = 0.051, BF_{10} = 0.225); late-cue vs. double-cue: t (22) = 2.067, *p* = 0.051, Cohen's d = 0.431, BF_{10} = 1.307).

SD

The average SD scores for the color report trials and the orientation report trials are illustrated in SFigure 2C and SFigure 2D, respectively.

Results of the repeated measures ANOVA for color report trials revealed no significant main effect of cue type, F (3, 66) =0.095, p = 0.963, η_p^2 = 0.004. However,

results of the ANOVA for orientation report trials showed significant main effect of cue type, F (3,66) = 3.806, p = 0.014, $\eta_p^2 = 0.147$.

The dimension-based single retro-cue benefit

For color report trials, planned comparisons revealed that participants' SD were no significant difference between the single-cue trials and the baseline (early-cue vs. neutral-cue: t (22) = 0.164, *p* = 0.871, Cohen's d = 0.034, BF_{10} = 0.221; late-cue vs. neutral-cue: t (22) = 0.172, *p* = 0.865, Cohen's d = 0.036, BF_{10} = 0.222).

Comparably, for orientation report trials, planned comparisons revealed significantly difference late-cue trials and neutral-cue trials, t (22) = 2.223, *p* = 0.037, Cohen's d = 0.463, BF_{10} = 1.684, but no difference between the early-cue trials and the neutral-cue, t (22) = 1.817, *p* = 0.083, Cohen's d = 0.379, BF_{10} = 0.897.

Furthermore, participants' SD for each dimension in single-cue conditions were independent from the onset of valid retro-cues (early-cue vs. late-cue, color: t (22) = 0.715, *p* = 0.482, Cohen's d = 0.149, BF_{10} = 0.276; early-cue vs. late-cue, orientation: t (22) = 0.679, *p* = 0.504, Cohen's d = 0.142, BF_{10} = 0.269).

The dimension-based double retro-cue benefit

Participants' SD didn't show difference between in trials with double cues and in trials with a neutral cue, regardless of the probed dimension (color: t (22) = 0.237, *p* = 0.815, Cohen's d = 0.049, BF_{10} = 0.224; orientation: t (22) = 0.106, *p* = 0.917, Cohen's d = 0.022, BF_{10} = 0.220).

The comparison of double-cue and single-cue conditions

The performance for colors did not differ between valid cue types (early-cue vs. double-cue: t (22) = 0.597, *p* = 0.557, Cohen's d = 0.124, BF10 = 0.257; late-cue vs. double-cue: t (22) = 0.095, *p* = 0.925, Cohen's d = 0.020, BF10 = 0.220).

However, the SD for orientations showed significantly difference between valid cue types (early-cue vs. double-cue: t (22) = 2.274, *p* = 0.033, Cohen's d = 0.474, BF10 = 1.836); late-cue vs. double-cue: t (22) = 3.228, *p* = 0.004, Cohen's d = 0.673, BF10 = 10.975).

Non-target report rate

The average non-target report rate for the color report trials and the orientation report trials are illustrated in SFigure 2E and SFigure 2F, respectively.

Results of the repeated measures ANOVA for color report trials revealed a significant

main effect of cue type, F (3, 66) =3.873, p = 0.013, η_p^2 = 0.150. The results of the ANOVA for orientation report trials revealed a similar pattern as those for color report trials, showing significant main effect of cue type, F (3, 66) = 4.668, p = 0.005, η_p^2 = 0.175.

The dimension-based single retro-cue benefit

For color report trials, planned comparisons revealed that participants' non-target report rate was significantly lower in the late-cue trials than in the neutral-cue trials, *t* (22) = 2.774, *p* = 0.011, Cohen's d = 0.578, BF_{10} = 4.505, but no difference between early-cue trials and neutral-cue trials, *t* (22) = 1.431, *p* = 0.167, Cohen's d = 0.298, BF_{10} = 0.535.

Comparably, for orientation report trials, planned comparisons revealed that participants' non-target report rate was significantly lower in the late-cue trials than in the neutral-cue trials, *t* (22) = 2.510, *p* = 0.020, Cohen's d = 0.523, BF_{10} = 1.034, but no difference between early-cue trials and neutral-cue trials, *t* (22) = 1.916, *p* = 0.068, Cohen's d = 0.399, BF_{10} = 2.771.

Furthermore, participants' non target report rate in single-cue conditions were independent from the onset of valid retro-cues for orientation, *t* (22) = 1.185, *p* = 0.249, Cohen's d = 0.247, BF_{10} = 0.407. However, there was significantly difference between early-cue and late-cue for color dimension, *t* (22) = 2.625, *p* = 0.015, Cohen's d = 0.547, BF_{10} = 3.415.

The dimension-based double retro-cue benefit

Participant's non-target report rate lower in trials with double cues than in trials with a neutral cue for orientation dimension *t* (22) = 2.902, *p* = 0.008, Cohen's d = 0.605, BF_{10} = 5.753, not for color dimension, *t* (22) =1.791, *p* = 0.087, Cohen's d = 0.373, BF_{10} = 0.861.

The comparison of double-cue and single-cue conditions

The performance for colors did not differ between valid cue types (early-cue vs. double-cue: *t* (22) = 0.683, *p* = 0.502, Cohen's d = 0.142, BF10 = 0.270; late-cue vs. double-cue: *t* (22) = 1.530, *p* = 0.140, Cohen's d = 0.319, BF10 = 0.605).

Meanwhile, the performance for orientations did not differ between valid cue types (early-cue vs. double-cue: *t* (22) = 1.839, *p* = 0.079, Cohen's d = 0.384, BF10 = 0.923; late-cue vs. double-cue: *t* (22) = 0.208, *p* = 0.837, Cohen's d = 0.043, BF10 =0.223).

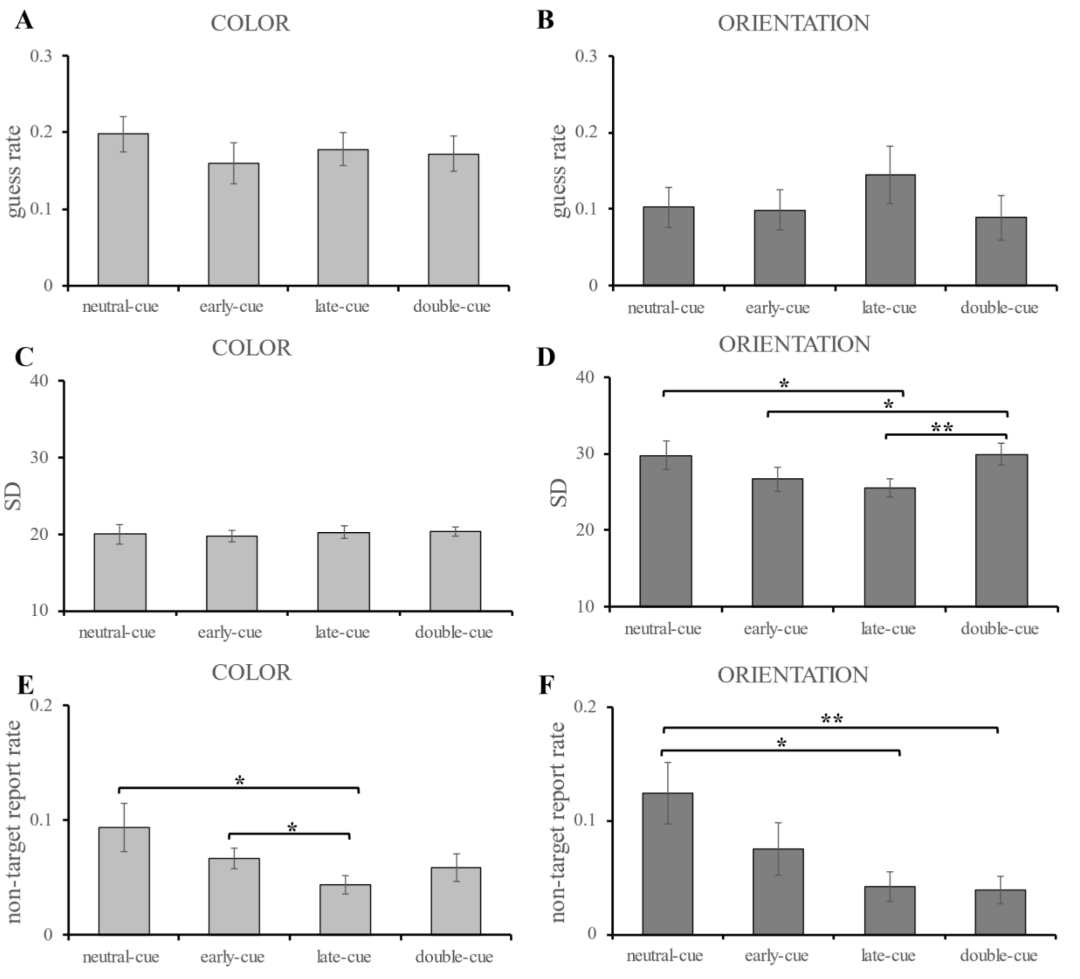

SFigure 1 Guess rate, SD, Non-target report rate results of Experiment 2. A higher bar with larger reproduction errors reflects a worse performance in VWM task. Light gray bars represent mean reproduction errors for color report trials (A, C, E). Dark gray bars represent mean reproduction errors for orientation report trials (B, D, F). Error bars indicate SE. * = $p < 0.050$, ** = $p < 0.01$.

Discussion

In the results of reproduction errors, we consistently observed a dimension-based retro-cue benefit across various cue types and dimension conditions. The results of the non-target report rate revealed a retro-cue benefit for both color and orientation dimensions under the double-cue and late-cue conditions, with the benefit in the color dimension under the double-cue condition being marginally significant. No retro-cue benefit was found in the guess rate. For the memory precision (SD), no retro-cue benefit in was observed for the color dimension, but a late-cue benefit was present for the orientation dimension. We suggest that the observed dimension retro-cue benefit is associated with a lower non-target report rate and improved precision (specifically for the orientation dimension), rather than a reduction in guess rate. Additionally, the non-

target report rate demonstrated a consistent pattern of no performance reduction under the double-cue condition compared to the single-cue condition. This suggests that the initial early cue did not exclude the unfocused dimension from VWM but rather prioritized the focused dimension.

The different pattern observed in the SD results may be attributed to differences in the consolidation processes between the two dimensions. Individuals typically use parallel consolidation for colors and serial consolidation for orientations (Hao et al., 2018; Liu & Becker, 2013; Miller et al., 2014). A retro-cue directed at the color dimension did not significantly improve memory precision because participants inherently consolidate color information rapidly. Due to this quick consolidation process, the colors of the three items were likely already stored in VWM with high precision before the retro-cue appeared. The role of the cue was thus to facilitate the prioritized retrieval of the cued color dimension, meaning that whether or not a color was the focus did not affect its storage precision. In contrast, the consolidation of orientations is a more time-consuming process, and an early retro-cue directed at this dimension may allocate additional resources to orientation, thereby speeding up consolidation and enhancing precision. Moreover, previous experimental findings suggest that retro-cues have a weaker impact on the precision of color dimensions compared to their effect on orientation dimensions (Park et al., 2017; Ye et al., 2016). Although the patterns in previous studies do not align perfectly with our findings, they do provide partial support for our results.

Interestingly, we found that memory precision under the double retro-cue condition was lower than under both the early-cue and late-cue conditions for the orientation dimension. To fully understand this result, it is important to consider the non-target report rate. Although the memory precision for orientation under the double-cue condition was lower than under the single-cue condition and did not significantly differ from the neutral cue condition, the non-target report rate was lower than in both the single-cue and neutral cue conditions. This suggests that, although the first retro-cue did not specifically target the orientation dimension, participants inadvertently engaged in low-precision consolidation of orientation, which was then rapidly retrieved upon presentation of the second retro-cue targeting orientation. The prior consolidation of orientation, despite its low precision, led to a significant reduction in the non-target report rate for the orientation dimension when the second retro-cue was presented, compared to the neutral condition. This implies that, having completed the initial storage process, participants were able to allocate more resources to decision-making, thereby decreasing the non-target report rate. These findings support our conclusion that the unfocused dimension in VWM is not impaired.

The distribution of reported colors as a function of the actual colors

In our study, we utilized a color wheel composed of colors that linearly changed in saturation (RGB) at each step, following the methodology in Ye et al. (2016). The RGB value was assigned as follows:

$[255, 75 + 3 \times n, 75]$ for $< = 0$ $n < = 59$

$[255 - 3 \times (n - 60), 255, 75]$ for $60 < = n < = 119$

$[75, 255, 75 + 3 \times (n - 120)]$ for $120 < = n < = 179$

$[75, 255 - 3 \times (n - 180), 255]$ for $180 < = n < = 239$

$[75 + 3 \times (n - 240), 75, 255]$ for $240 < = n < = 299$

$[255, 75, 255 - 3 \times (n - 300)]$ for $300 < = n < = 359$

Reference

Bays, P. M., Catalao, R. F. G., & Husain, M. (2009). The precision of visual working memory is set by allocation of a shared resource. *Journal of Vision*, *9*(10), 7.1-11.

Faul, F., Erdfelder, E., Lang, A.-G., & Buchner, A. (2007). G*Power 3: A flexible statistical power analysis program for the social, behavioral, and biomedical sciences. *Behavior Research Methods, 39*(2), 175–191.

Hao, R., Becker, M. W., Ye, C., Liu, Q., & Liu, T. (2018). The bandwidth of VWM consolidation varies with the stimulus feature: Evidence from event-related potentials. J Exp Psychol Hum Percept Perform, 44(5), 767-777

Liu, T., & Becker, M. W. (2013). Serial consolidation of orientation information into visual short-term memory. Psychol Sci, 24(6), 1044-1050.

Miller, J. R., Becker, M. W., & Liu, T. (2014). The bandwidth of consolidation into visual short-term memory depends on the visual feature. Vis cogn, 22(7), 920-947.

Suchow, J. W., Brady, T. F., Fougnie, D., & Alvarez, G. A. (2013). Modeling visual working memory with the MemToolbox. Journal of Vision, 13.

Park, Y. E., Sy, J. L., Hong, S. W., & Tong, F. (2017). Reprioritization of Features of Multidimensional Objects Stored in Visual Working Memory. Psychological science, 28(12), 1773–1785.

Ye, C., Hu, Z., Ristaniemi, T., Gendron, M., & Liu, Q. (2016). Retro-dimension-cue benefit in visual working memory. *Scientific Reports*, *6*, 35573.

Zhang, W., & Luck, S. J. (2008). Discrete fixed-resolution representations in visual working memory. *Nature, 453*(7192), 233–235.
